# A new species of *Pseudomphala* Heude, 1882 (Gastropoda, Truncatelloidea, Assimineidae) from Zhoushan of Zhejiang Province, East China Sea

**DOI:** 10.3897/zookeys.1283.181408

**Published:** 2026-06-24

**Authors:** Wenliang Liu, Feijun Zhang, Liu Tao, Jiyao Li, Xiaosong Li

**Affiliations:** 1 School of Ecological and Environmental Science, East China Normal University, Shanghai, 200241, China School of Ecological and Environmental Science, East China Normal University Shanghai China; 2 Zhoushan Workstation, Ningbo Ocean Center, Ministry of Natural Resources, Zhoushan, Zhejiang, 316022, China Zhoushan Workstation, Ningbo Ocean Center, Ministry of Natural Resources Zhoushan China; 3 Zhoushan Marine Environment Monitoring and Forecasting Center, Zhoushan, Zhejiang, 316022, China Zhoushan Marine Environment Monitoring and Forecasting Center Zhoushan China

**Keywords:** Assimineinae, COI, mitochondrial morphology, *Pseudomphala zhoushanensis*, taxonomy

## Abstract

A new species that is putatively assigned to the genus *Pseudomphala* Heude, 1882 (Truncatelloidea, Assimineidae) and that was collected from the East China Sea is described and illustrated. The new species is characterized by: a shell with subsutural thread, umbilicus covered, a penis without papilla, central teeth of the radula with 4–5 pairs of strong basal cusps laterally, and outer marginal teeth with 6–7 large cusps and a thin flaring flap. Furthermore, Bayesian inference and maximum-likelihood analyses conducted on a partial sequence of the COI mitochondrial fragment have provided additional support for the systematic classification of this new taxon.

## Introduction

The family Assimineidae H. Adams & A. Adams, 1856 is regarded as one of the most evolutionarily derived groups within the truncatelloidean gastropods (Ponder 1988). The family exhibits a broad geographical distribution, occurring across both temperate and tropical regions (Marcus and Marcus 1965; Abbott 1974; van Aartsen 2008). In the East China Sea, only three Assimineidae species have been recorded so far: *Assiminea lutea* A. Adams, 1861, *A. violacea* Heude, 1882 and *Pseudomphala latericea* (H. Adams & A. Adams, 1864) (Cai and Huang 1991). In a more recent analysis, Fukuda (2019) regarded *A. violacea* as a synonym of *A. grayana* J. Fleming, 1828, based on their anatomical and DNA sequence similarities.

In the context of a comprehensive investigation of the marine fauna of Zhoushan City, located in Zhejiang Province along the East China Sea, an undescribed putative *Pseudomphala* Heude, 1882 species was found on an intertidal mud flat. *Pseudomphala* is characterized by the following characters: ovate-conical to conical shell; subsutural thread distinct; operculum with elongated posterior end; pentagonal central radular tooth with strong basal cusps and a large, long projection on the ventral margin; and outer marginal teeth with fewer than 10 cusps (Fukuda and Mitoki 1996). Two species are known, both from the northwestern Pacific: *P. latericea* (China and the Korean Peninsula) (Yuan et al. 2025) and *P. miyazakii* (T. Habe, 1943) (endemic to Kyushu, Japan) (Fukuda and Mitoki 1996).

In this contribution, we describe and illustrate the new species by examining its shell morphology and aspects of the internal anatomy. Furthermore, we conducted a molecular phylogenetic analysis of the new species alongside other taxa within Assimineidae, utilizing partial sequences of the mitochondrial cytochrome *c* oxidase subunit I (COI) gene.

## Material and methods

### Sample collection and morphological analysis

All specimens were collected from the coastal intertidal zone and preserved in 80% ethanol. Live animals were removed from the shells without damage by applying the niku-nuki method, which involves boiling the specimens in water at 90–95 °C for 3 s (Fukuda et al. 2008). This procedure was easy to perform and was successful in all specimens examined. Muscle tissue (part of the foot and columellar muscle) was then removed from the soft parts and fixed in 99% ethanol for further DNA analysis. The remaining soft parts were fixed in 5% neutral seawater formalin (hereafter SWF) for anatomical examination. Radulae were extracted following the methods described by Fukuda and Mitoki (1995). Photographs were made with a Zeiss V16 stereomicroscope and Phenom Pure^+^ scanning electron microscope (SEM). The shell length, shell width, aperture length, aperture width, and body whorl length of selected specimens were measured with a vernier caliper. The specimens were deposited in the Yangtze River Delta Estuary Wetland Station, Shanghai, China (YARDEWS).

### DNA sequencing

Total genomic DNA was extracted from the foot muscle of the specimens using a TIAN amp Marine Animals DNA Kit (TIANGEN) according to the manufacturer’s instructions. The target DNA fragment of the COI gene was amplified by polymerase chain reaction (PCR) with the following primers: LCO1490 5^’^- GGTCAACAAATCATAAAGATATTGG -3^’^ and HCO2198 5^’^- TAAACTTCAGGGTGACCA AAAAATCA -3^’^ (Folmer et al. 1994). To resolve potential confusion due to sexual dimorphism, one male and one female individual were selected from the paratype specimens for molecular analysis. PCR was performed following the protocol: a total volume of 20 µl consisting of 10 * EX Taq Buffer (Mg^2+^ Plus), 2.5 mM dNTPs, 0.5 µM of primers LCO1490 and HCO2198, 5U Takara Ex Taq, 1 µl (total amount of DNA < 200 ng) DNA extract and ddH_2_O. The PCR cycle was programmed as follows: 94 °C for 1 min, 37 cycles of 94 °C for 30 s, 50 °C for 30 s, and 72 °C for 1 min, with a final extension step at 72 °C for 10 min, followed by storage at 4 °C. The PCR was performed as a touchdown PCR. All PCR products were purified and sequenced by the Beijing Genomics Institute. DNA sequence chromatograms were checked for errors and edited with DNASTAR Lasergene 7.1.0 (DNASTAR Inc., Madison, WI, USA). COI gene sequences were aligned using CLUSTAL W (Thompson et al. 1994).

Methods used for molecular phylogenetic analysis included maximum likelihood (ML) and Bayesian inference (BI). The TIM3+I+G model of sequence evolution (Posada 2008) was selected based on the Akaike Information Criterion (Akaike 1973) using jModelTest v. 2.1.10 (Darriba et al. 2012). Due to limitations in the MEGA v. 11.0.13 (Tamura et al. 2021) model library, the ML analysis employed TrN+I+G (Tamura and Nei 1993) as the closest approximation to the optimal model, with 5000 thorough bootstrap replicates. A consensus tree with support indices was generated by MrBayes v. 3.2.7 (Ronquist et al. 2012) using the TIM3+I+G model of sequence evolution (Posada 2008). MrBayes employs Markov chain Monte Carlo (MCMC) methods, implemented by running four Markov chains for 4 million generations each, sampling every 100 generations. The first 25% of trees were conservatively discarded as burn-in, and stationarity was confirmed by examining the log-likelihood plot in Tracer (Rambaut and Drummond 2007). Trees were visualized using FigTree v. 1.4.5. Nodal support was considered high for Bayesian posterior probabilities ≥ 0.95 and bootstrap values ≥ 80%, and moderate for Bayesian posterior probabilities between 0.85 and 0.94 and bootstrap values between 70% and 79%. Nodes with lower support values were not considered significant (Hallan et al. 2015). A phylogram based on COI was generated using 19 sequences from the family Assimineidae, of which 16 sequences were retrieved from GenBank. A COI sequence from *Littorina brevicula* (R. A. Philippi, 1844), obtained from GenBank, was used as the outgroup (Table 1). Sequences of *Pseudomphala zhoushanensis* Liu, sp. nov. and *Pseudomphala latericea* were newly generated in this study.

**Table 1.** List of specimens used for molecular analysis, with date and collection site, voucher number and GenBank accession numbers.

**Species**	**Collection site**	**Vouchers**	**GenBank accession No. (COI)**
*Pseudomphala zhoushanensis* Liu, sp. nov.	Zhoushan, Zhejiang, China	20240421-DS-1C/8 (= paratype, male)	PQ608369
		20240711-XGD-1C/2 (=paratype, female)	PQ608371
*Pseudomphala latericea* (H. Adams & A. Adams, 1864)	Zhoushan, Zhejiang, China	20240331-1	PQ607811
*Pseudomphala miyazakii* (T. Habe, 1943)	Saga, Japan	personal:Kame da Y.:1920	AB611815
*Angustassiminea andrewsiana* (E.A. Smith, 1900)	Christmas Island, Australia	C.453054	HG973051
*Angustassiminea californica* (Tryon, 1865)	San Pablo Bay, USA	A1A	DQ533855
*Angustassiminea satumana* (T. Habe, 1942)	Kagoshima, Japan	Kameda Y: 5382	AB611803
*Assiminea estuarina* T. Habe, 1946	Korea	NSMK-MS- 150300129	OL877322
*Assiminea grayana* J. Fleming, 1828	Medway Estuary, UK	USNM1096073	EF667310
*Cavernacmella kuzuuensis* K. Suzuki, 1937	Yoron Island, Japan	CcY	AB822675
*Cavernacmella minima* T. Habe, 1942	Ogasawara Islands, Japan	Cc9	AB822671
*Conassiminea zheni* H. Fukuda & Ponder, 2006	Blind Bight, Australia	AH-As118	LN680854
*Cryptassiminea buccinoides* (Quoy & Gaimard, 1834)	Bittern, Australia	C.404658	HG973049
*Cryptassiminea tasmanica* (Tenison-Woods, 1876)	Careel Bay, Australia	C.415219	HG973050
*Ovassiminea annulata* Hallan, H. Fukuda & Kameda, 2015	Cobourg Peninsula, Australia	AH-2014 2	LN680860
*Ovassiminea miskellyi* Hallan, H. Fukuda & Kameda, 2015	Hanover Bay, Australia	AH-2014 1	LN680859
*Suterilla julieae* H. Fukuda, Ponder & B.A. Marshall, 2006	Tatlows Beach, Tasmania	AMS C.466936	LN680863
*Taiwanassiminea affinis* (O. Boettger, 1887)	Wisemans Ferry, Australia	C.269583	HG973054
*Taiwanassiminea phantasma* Hallan & H. Fukuda, 2015	Middle Osborn Island, Australia	C.461139	HG973055
*Littorina brevicula* (R.A. Philippi, 1844)	Dalian, China	LB20190611	MT362562

## Taxonomy

### Family Assimineidae H. Adams & A. Adams, 1856

**Subfamily Assimineinae (= ‘ Group 1 ’ sensu Fukuda and Ponder 2003)**

#### Pseudomphala zhoushanensis

Taxon classificationAnimaliaLittorinimorphaAssimineidae

Liu sp. nov.

3340BA11-D8C4-50CA-BB1E-8482EED346B9

https://zoobank.org/453988ED-84D6-45A0-BFC5-2E9367A91345

**Figure 1.**
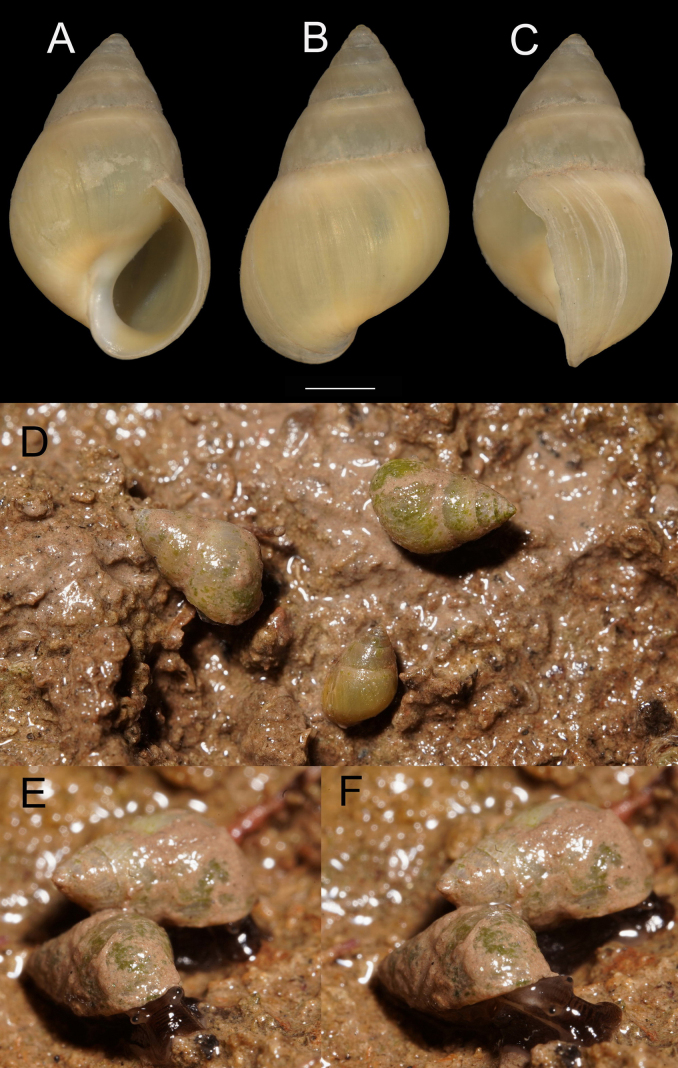
*Pseudomphala zhoushanensis* Liu, sp. nov. **A–C**. Holotype 20240421-DS-1C/1 **D**. Paratypes 20240421-DS-1C/2–4; **E, F**. Paratypes 2024042-DS-1C/5–6. **A–C**. Shell (ventral, dorsal and lateral view); **D–F**. Live specimen. Scale bars: 1 mm.

**Figure 2.**
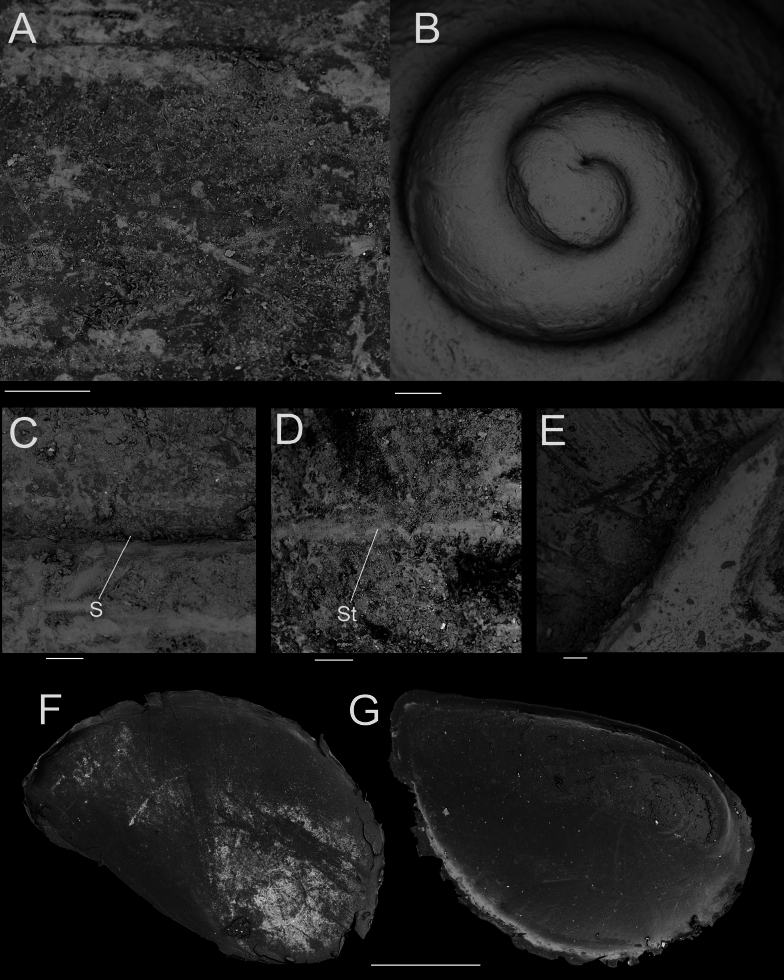
*Pseudomphala zhoushanensis* Liu, sp. nov. paratype 20240421-DS-1C/7. **A**. Surface of shell; **B**. Protoconch; **C**, **D**. Suture and subsutural thread; **E**. Umbilical area; **F, G**. Operculum (outer and inner view). Abbreviation: S: suture, St: subsutural thread. Scale bars: 50 µm (**A–E**); 500 µm (**F, G**).

**Figure 3.**
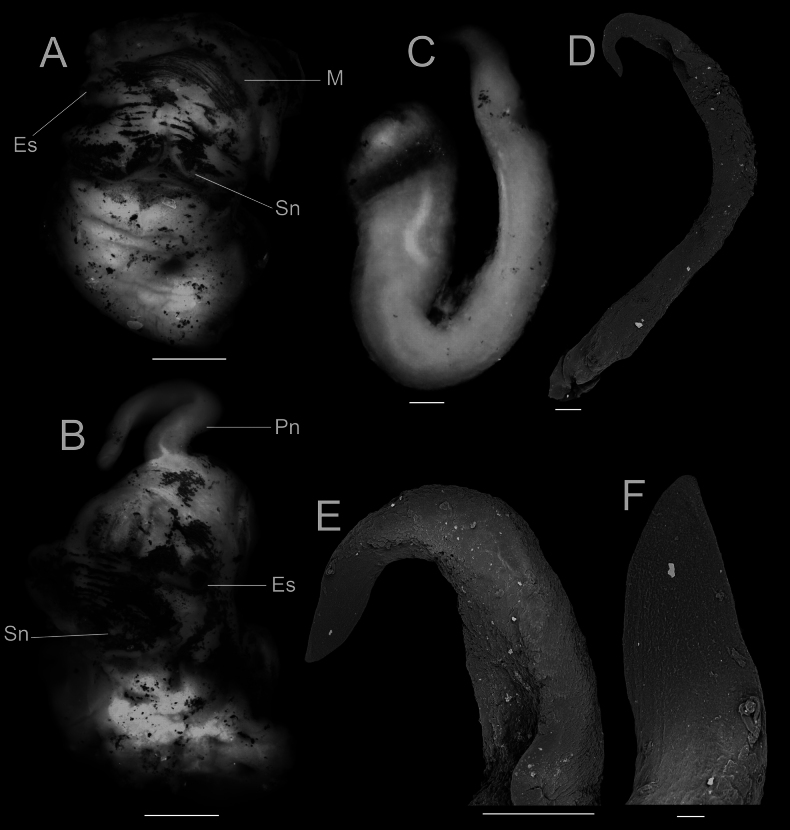
*Pseudomphala zhoushanensis* Liu, sp. nov. paratype 20240421-DS-1C/7(male). **A, B**. Head-foot; **C**. Penis; **D–F**. Penis (SEM). Abbreviation: Es: eye stalk, M: mantle, Pn: penis, Sn: snout. Scale bars: 500 µm (**A, B**); 100 µm (**C–E**); 10 µm (**F**).

**Figure 4.**
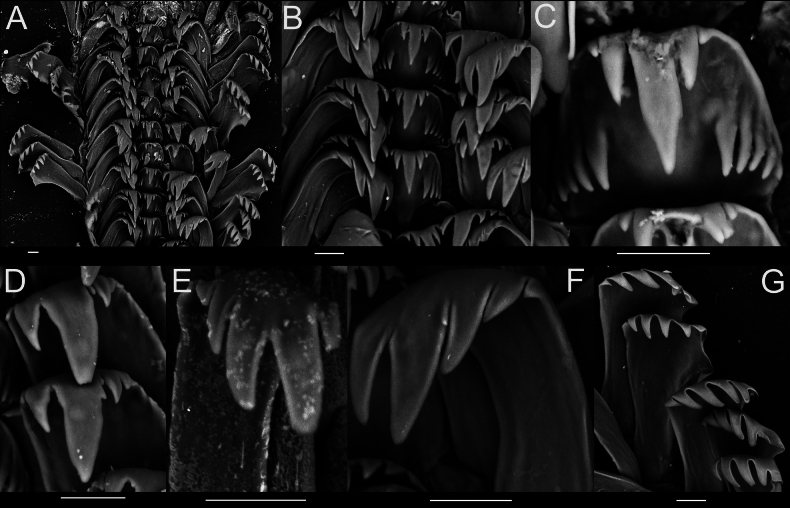
*Pseudomphala zhoushanensis* Liu, sp. nov. paratype 20240421-DS-1C/7. **A**. Portion of radula ribbon; **B**. Central and lateral teeth; **C**. Central teeth; **D**. Lateral teeth; **E, F**. inner marginal teeth; **G**. Outer marginal teeth. Scale bars: 50 µm (**A**); 10 µm (**B–G**).

**Figure 5.**
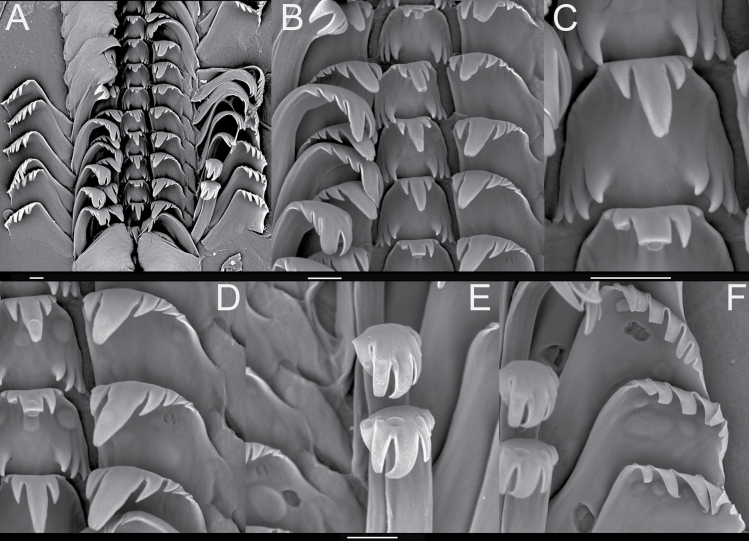
*Pseudomphala zhoushanensis* Liu, sp. nov. paratype 20240711-XGD-1C/1. **A**. Portion of radula ribbon; **B**. Central and lateral teeth; **C**. Central teeth; **D**. Lateral and central teeth; **E**. Inner marginal teeth; **F**. Inner and outer marginal teeth. Scale bars: 10 µm (**A–F**).

**Figure 6.**
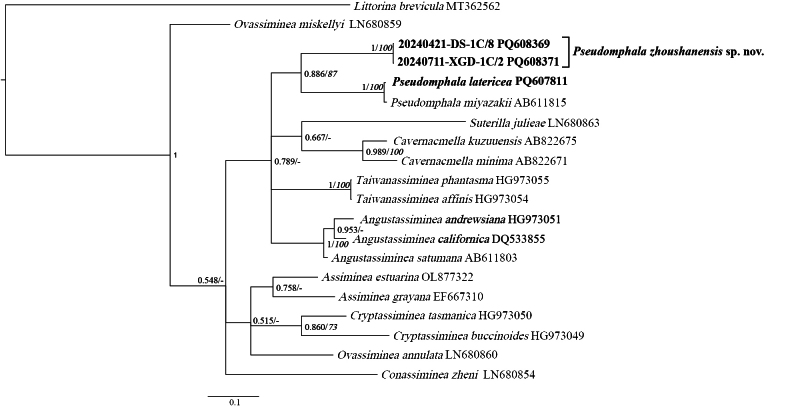
COI Bayesian tree, showing the phylogenetic relationship of *Pseudomphala zhoushanensis* Liu, sp. nov. GenBank codes are provided for each COI sequence. BI posterior probability values of > 0.50 (left) and ML bootstrap indices of > 70 (right) are indicated by branch nodes. Species names follow the original identifications of GenBank sequences. Sequences obtained in this study are shown in bold.

Figs 1, 2, 3, 4, 5, 6

##### Material examined.

***Holotype***: 20240421-DS-1C/1, Daishan, Zhoushan, Zhejiang Province, 30°18.555'N, 122°6.180'E, intertidal zone, soft mud, coll. Xiaosong Li, 21 April 2024. ***Paratypes***: type locality, 13 specimens, 20240421-DS-1C/2–14 • 6 specimens, 20240711-XGD-1C/1–6, Xiaogan Island, Zhoushan, Zhejiang Province, 29°58.485'N, 122°11.898'E, intertidal zone, soft mud, coll. Xiaosong Li, 11 July 2024.

##### Other material.

7 specimens, 20240531-DS-1C, type locality, intertidal zone, soft mud, coll. Xiaosong Li, 31 May 2024 • 15 specimens, 20241012-XGD-1C, Xiaogan Island, Zhoushan, Zhejiang Province, 29°58.485'N, 122°11.898'E, intertidal zone, soft mud, coll. Xiaosong Li, 12 October 2024.

##### Diagnosis.

Shell of mature individuals ovate-conical to conical, subsutural thread extremely thin. Umbilicus covered. Operculum pyriform, transparent, with eccentric nucleus. Head-foot brown. Penis without papilla. Central radular teeth with 5 strong cusps, median cusps longest; 4–5 pairs of strong basal cusps laterally. Lateral teeth with 7 strong cusps. Inner marginal teeth with 5–6 sharp cusps. Outer marginal teeth with 6–7 large cusps and with thin, flaring flap.

##### Description.

***Shell*** (Fig. 1A–F) small, ovate-conical to conical, thin but solid, shiny and opaque; color uniformly yellow-green to greyish-green. Protoconch (Fig. 2B) with 2.0–2.5 very weakly convex whorls, outer surface without sculpture. Teleoconch with 2.0–5.0 weakly convex whorls, rough growth lines and microscopic spiral threads found irregularly on surface (Fig. 2A); suture very shallow (Fig. 2C), subsutural thread extremely thin (Fig. 2D). Body whorl large, occupies about 1/2 to 3/5 of whole length. Aperture wide, pyriform; peristome complete, sharp; outer lip slightly expanded outwardly; inner lip rather long, broad, thick callus on body whorl; columellar lip continued from inner lip, thick, gently curved. Umbilical area (Fig. 2E) narrow, distinctly excavated, with many distinct, dense growth lines; umbilicus absent. Measurements: see Table 2. Shell length up to 6.40 mm, shell width up to 3.58 mm.

**Table 2.** Shell measurements (mm) of *Pseudomphala zhoushanensis* Liu, sp. nov.

**Vouchers (*N*)**	**Shell length**	**Shell width**	**Aperture length**	**Aperture width**	**Body whorl length**
20240421-DS-1C (15)	4.18 ± 0.76 (4.83, 2.74)	2.20 ± 0.29 (2.62, 1.65)	2.10 ± 0.23 (2.46, 1.67)	1.77 ± 0.18 (2.07, 1.43)	3.02 ± 0.46 (3.59, 2.14)
20240531-DS-1C (7)	4.94 ± 0.70 (6.40, 3.84)	2.82 ± 0.36 (3.43, 2.13)	2.50 ± 0.24 (2.83, 2.03)	2.01 ± 0.24 (2.41, 1.70)	3.62 ± 0.44 (4.44, 2.91)
20240711-XGD-1C (6)	5.04 ± 0.21 (5.41, 4.74)	2.99 ± 0.32 (3.38, 2.64)	2.74 ± 0.36 (3.34, 2.37)	2.23 ± 0.43 (3.15, 1.87)	3.79 ± 0.17 (4.05, 3.54)
20241012-XGD-1C (15)	5.37 ± 0.35 (5.97, 4.31)	3.21 ± 0.29 (3.58, 2.51)	2.65 ± 0.20 (2.86, 2.10)	2.06 ± 0.17 (2.41, 1.74)	3.90 ± 0.21 (4.15, 3.41)
Total (43)	4.84 ± 0.76 (6.40, 2.74)	2.76 ± 0.54 (3.58, 1.65)	2.45 ± 0.36 (3.34, 1.67)	1.98 ± 0.29 (3.15, 1.43)	3.53 ± 0.52 (4.44, 2.14)

Holotype: 20240421-DS-1C/1; paratypes: specimens from 20240421-DS-1C/2–14 and 20240711-XGD-1C/1–6.

***Operculum*** (Fig. 2F, G) pyriform, paucispiral, thin, horny, yellowish, transparent, and nucleus offset from centre; muscle scar elongate, extending along most of inner surface.

***Anatomy***. Mantle, head-foot and eye stalks brown (Figs 1D, 1E, 3A, 3B). Eye stalks short and blunt; eyes situated in middle of eye stalk. Snout moderately long, bilobed. Foot large, anterior edge horizontal or slightly rounded, distal end tapering. Dioecious, male penis (Fig. 3C–F) rather slender, counter-clockwise, attached to near middle of head well behind eye stalks, simple, without fringe; tip pointed, without penial papilla.

***Radula*** taenioglossate (Figs 4, 5). Central teeth with 5 strong cusps, median cusps longest, up to twice as long as adjacent cusps, tip sharp; 4–5 pairs of strong basal cusps laterally (Figs 4B, 4C, 5B–D). Lateral teeth with 7 strong cusps (Figs 4D, 5D), median cusp wide and long, tip sharp, armed with 1–2 minute accessional cusps on the outer margin occasionally (Fig. 4D), lateral cusps short, triangular and curved. Inner marginal teeth distinctly curved distally (Figs 4E, 4F, 5E, 5F), with 5–6 sharp cusps. Outer marginal teeth wide, curved distally (Figs 4G, 5F), with 6–7 sharp cusps and with a thin flaring flap.

##### COI sequence results.

We sequenced a portion of the COI gene of the new taxon and submitted the sequences to the National Center for Biotechnology Information (NCBI) GenBank database. The accession numbers of two sequences are PQ608369 and PQ608371. After alignment and trimming, 564 bp sequences were obtained from 20 individuals. The resulting tree topology was consistent with the Bayesian inference shown in Fig. 6. According to this phylogeny, both BI and ML analyses showed that the two specimens of *Pseudomphala zhoushanensis* Liu, sp. nov. shared the same COI sequence, and *Pseudomphala latericea* and *Pseudomphala miyazakii* were moderately supported (PP = 0.886, ML = 87%) as sister to the new taxon, forming a polytomy with other genera (PP = 0.789) (Fig. 6).

##### Etymology.

The species name is derived from the type locality, Zhoushan City.

##### Distribution and habitat.

Presently only known from the type locality. Both our survey data and historical records suggest that it does not occur in the neighboring regions, such as Shanghai and Fujian Province. It inhabits the soft mud in the intertidal zone and creeps on the base of smooth cordgrass (*Sporobolus alterniflorus*) marsh.

##### Remarks.

Among the currently known taxa of Assimineidae, *Pseudomphala zhoushanensis* Liu, sp. nov. can be assigned to ‘Group 1’ sensu Fukuda and Ponder (2003) (= subfamily Assimineinae) because (1) its central radular teeth have multiple basal cusps and (2) it possesses very indistinct cephalic tentacles. The genus *Pseudomphala* has only two species, *P. latericea* and *P. miyazakii*. *Pseudomphala zhoushanensis* Liu, sp. nov. is assigned to *Pseudomphala* on account of the ovate-conical to conical shell; subsutural thread distinct; operculum with elongated posterior end; pentagonal central radular tooth with strong basal cusps and a large, long projection on the ventral margin; and outer marginal teeth with fewer than 10 cusps. Yet, the assignment of the new species to *Pseudomphala* is not straightforward, as the radular teeth morphology of *P. zhoushanensis* Liu, sp. nov. is distinct from that of the other two *Pseudomphala* species, *P. latericea* and *P. miyazakii*, since its central teeth have 4–5 pairs of strong basal cusps (vs 3 pairs of short basal cusps in the two other species) and its outer marginal teeth have a thin flaring flap (vs no flap in the two other species). Moreover, *P. zhoushanensis* Liu, sp. nov. lacks a penial papilla, whereas the other two species have a distinct one. The new species also shows considerable similarities to species of *Angustassiminea* T. Habe, 1943, especially regarding the shell shape, the presence of a subsutural thread on the shoulder, and the lack of a penial papilla (Fukuda and Mitoki 1996). Nevertheless, the new species can be distinguished from *Angustassiminea* sp. by the shape of the radular teeth: the outer marginal teeth of the new species have only 6–7 large cusps, whereas those of *Angustassiminea* have usually more than 10 small cusps.

## Discussion

COI sequence comparison shows that *Pseudomphala zhoushanensis* Liu, sp. nov. is distinct from other species of the family Assimineidae for which COI sequences are available. Meanwhile, *P. latericea* and *P. miyazakii* were supported (PP = 0.886, ML = 87%) as sister taxon to the new species (Fig. 6). Given that the COI fragment is effective for species delimitation (Hebert et al. 2004), and considering the morphological data presented here, it is clear that *P. zhoushanensis* Liu, sp. nov. is a well-defined new species that shows similarities with *Pseudomphala* or *Angustassiminea*. Yet, despite these morphological similarities, the new species does not perfectly align with either of these two genera. Therefore, one might be tempted to erect a new genus for it. However, the establishment of monotypic genera remains controversial. Its limitations lie primarily in the lack of comparative context among congeneric species, which may compromise taxonomic stability and increase the likelihood of subsequent revisions. Platnick (1976) noted that a genus containing only a single species has inherent limitations in information content, as it provides little insight into the relationships between that species and other potentially related taxa. Anyway, based on the available literature and comparative evidence (Fukuda and Mitoki 1996), both morphological and COI sequence data are insufficient to justify establishing a new genus, the more so since the current taxon sampling remains far too limited. Under these circumstances, we refrain from proposing a new genus for the present species. Instead, the new species is tentatively assigned to the genus *Pseudomphala*, and is described as *Pseudomphala zhoushanensis* Liu, sp. nov., named after its type locality in Zhoushan City. Future studies incorporating broader geographic sampling and additional DNA sequence data from other taxa within Assimineinae are needed to clarify interspecific relationships and to reassess generic boundaries within the group.

## Supplementary Material

XML Treatment for Pseudomphala zhoushanensis

## Additional information

### Artificial Intelligence (AI) use

The authors accept full responsibility for the content of the manuscript, including the disclosure of any use of AI.

No AI tools were used in the preparation of this manuscript.

### Author ORCIDs

W. Liu https://orcid.org/0000-0003-3964-4834

F. Zhang https://orcid.org/0009-0005-0255-4407

X. Li https://orcid.org/0009-0001-3773-0790

### Data availability

All of the data that support the findings of this study are available in the main text.
